# Social autopsy for maternal and perinatal deaths in Bangladesh: a tool for community dialog and decision making

**DOI:** 10.1186/s40985-018-0098-3

**Published:** 2018-07-05

**Authors:** Animesh Biswas, Junnatul Ferdoush, Abu Sayeed Md Abdullah, Abdul Halim

**Affiliations:** Centre for Injury Prevention and Research, Bangladesh (CIPRB), Dhaka, Bangladesh

**Keywords:** Social autopsy, Maternal and Perinatal Death Review System, Maternal and Perinatal Death Surveillance and Response, Verbal autopsy, Maternal and neonatal death, Bangladesh

## Abstract

Bangladesh has an established comprehensive death review system for tracking and reviewing maternal and perinatal deaths. This death review system, established in 2010, was initially known as the “Maternal and Perinatal Death Review (MPDR) System.” One of the key interventions of the MPDR system, social autopsy (SA), is generally undertaken following a maternal or perinatal death notification. Social autopsy is managed at the community level by government field health workers. The main purpose of SA is to enable community discussion and create awareness of the preventable causes of maternal or neonatal deaths. Through these conversations, it is hoped to reduce future maternal and neonatal deaths. During the scaling up of the system in Bangladesh in 2016, the Ministry of Health and Family Welfare (MoH&FW) included social autopsy as a useful intervention in reviewing death at the community level and named it “Maternal and Perinatal Death Surveillance and Response” (MPDSR). The new MPDSR tool is currently being administered for the the 2017 to 2021 period under the National Health and Nutrition Population Sector Program (HPNSP). This paper seeks to review the experiences of the social autopsy tool, from the initial MPDR system to the current MPDSR system and its role in reducing maternal and neonatal deaths in Bangladesh.

## Background

Social autopsy (SA) is an innovative tool, used worldwide, to determine the salient social determinants of death. There is much scientific research describing the remarkable achievements of SA in diminishing maternal and neonatal deaths, including stillbirths, in many low- and middle-income countries (LMIC) [1–3]. SA is an important community-oriented primary healthcare approach and plays an important role in addressing the social determinants of death [2]. The Maternal and Perinatal Death Inquiry and Response (MAPEDIR) approach empowered communities in India to reduce maternal deaths and also to design appropriate focused interventions [4, 5].

Being a LMIC, Bangladesh has experience of implementing SA as a community-based intervention in preventing death from injuries such as road traffic accident, drowning, and also death as a result of communicable disease [6–10]. In the light of past experiences, SA was introduced in 2010 through the partnership between the Centre for Injury Prevention and Research (CIPRB), Bangladesh, and the United Nations Children’s Fund (UNICEF), Bangladesh, in collaboration with the Government’s MPDR system. The SA initiative was initially piloted in Thakurgaon to alleviate maternal, neonatal deaths, and stillbirths [4, 5, 11–13]. After observing the positive effect in reducing stillbirths, maternal, and neonatal deaths, the Government of Bangladesh scaled it up to districts during the 2011 to 2012 period [5]. Positive results generated a potential background and platform to expand the system to 10 districts from 2013 to 2015, increasing to 14 districts of Bangladesh in 2015 [5]. The Government has now expanded the newly named “Maternal and Perinatal Death Surveillance and Response” (MPDSR) program in 22 districts and eventually plans to cover all districts in Bangladesh. SA has been incorporated in the MPDSR system as one of the key strategies to address avoidable maternal and neonatal deaths and thereby contribute to Bangladesh achievement of the 2030 Sustainable Developmental Goals (SDG): (3) to ensure healthy lives and promote well-being for all at all ages, specifically targets (3.1) to reduce maternal mortality, and (3.2) to end preventable deaths of newborns [11].

The system follows a cycle. When the government frontline health workers receive notification of a mother or newborn death, they conduct a verbal autopsy followed by SA [4]. SA is a unique, innovative, and effective approach when used in Bangladesh settings. As a successful community-based intervention, it may act as a model in other LMICs [12, 13]. Unlike SA in other countries, SA for maternal and perinatal death in Bangladesh does not focus on generation of data; rather, it explores the cause of death through community interaction and triggers community actions [12, 14–18]. This paper discusses the evolution of development and utilization of the SA tool and its role in reducing maternal and perinatal deaths in Bangladesh. The findings will be useful in the effective design of similar SA interventions for the improvement of maternal and perinatal health in countries with a similar context.

## Opportunities and challenges

### Process description of SA in maternal and neonatal death in the community

SA can help prevent death in the community by creating social awareness. The basic strategy is adopted from another intervention of child injury prevention in Bangladesh [9, 10]. In Maternal and Perinatal Death Surveillance and Response (MPDSR), SA is generally conducted following the completion of verbal autopsy and death review of a mother or neonate. The process is conducted by a government level frontline health worker’s committee, assigned by the local government-run Upazila Heath Complex and monitored by a district or national level monitoring committee. The community level grass root workers commence SA as soon as they get the notification from the community or health facility of a death or a serious non-fatal cause of injury to a mother or child [4]. Then, they conduct verbal autopsy to explore the medical causes of a death along with the associated factors and delays. After that, the committee organizes SA with between 40 and 50 members, including neighbors from adjacent 20 to 30 households, family members of the deceased, and senior community members. These latter are usually made up of community leaders, religious leaders, teachers, and members from the local government.

The SA meeting is usually held between 15 and 30 days after the death. SA facilitation takes place in the courtyard close to the home of the deceased. Usually, the meeting starts with neighbors expressing their view of what contributed to the death of the mother or newborn. With the revelation of the scenario, community people interact with each other and express their own views and opinions and gradually understand the deficiencies in their knowledge [18]. Community people discuss and prepare their own action plan along with the positive support from other valuable community members. Decisions made from the discussions typically include the assurance of at least four antenatal checkups, safe birth preparedness, and safe delivery for each pregnant mother in the community. It also ensures the prevention of all delays that could happen from the time of decision making till the end of referral to a facility. All of these decisions are implemented and monitored by community leaders along with the members of local government. Community members also make a commitment to try and prevent any other future deaths [19].

### Role of government health workers in facilitating SA

The Health Inspector, Family Planning Inspector or Sanitary Inspector from both Health and Family Planning Departments of the Government are the key actors in conducting the first line supervision (i.e., supervision of Health Assistant and Family Welfare Assistant) to arrange the SA at the community level. The health workers, with the help of community group members, neighbors, and family members of the deceased along with the support of other grassroots level health workers, organize a community level meeting. The health workers receive training on facilitation of the community session and develop skills to explore the social causes, stigma, and barriers held responsible for a death of a mother or neonate in the community. By encouraging the community people to speak in their own words, the health worker guides them to understand how events could have been handled better. The health worker also conveys important messages related to maternal or neonatal complications, birth planning, essential newborn care, antenatal care, postnatal care, and available facilities in the government hospital for the mother and neonate. They also show visual materials like flip charts, documentaries, and other important behavioral change communication materials to the local community. Many of these people are illiterate, so these simple visual aids can assist them in making future sound decisions. The health workers also seek commitment from the community about what actions they will take if similar maternal or neonatal complications arise in the future [12].

### Role of community people in a SA

The main target group of the SA meeting is the neighbors of the deceased. There is a special focus on the head of the household. These are mostly male and an important consideration of a SA session as they are the family decision makers. To ensure the involvement of male members of the deceased, SA usually takes place in early morning or late afternoon to keep working hours uninterrupted.

In a SA, around 40–50 participants gather, including the health worker as facilitator. During a session, community people discuss how the maternal or neonatal death occurred in their local neighborhood. Community members discuss the events that took place before the death of a mother or newborn such as health complications, health seeking behavior and delays in decision making, difficulties in transferring the patient, or delay in the provision of care at the facility level [11]. The forum finds out if there were any responsible social barriers, delays could have been prevented. They also talk about probable future solutions to prevent such unwanted death.

In each meeting, the health worker invites the community leader, who can be an elected person from local government, a school teacher, a religious leader, or a local elder to mobilize the society towards good practices. The presence of these people during the meeting helps to develop accountability for their community to act in the best possible way to prevent future maternal or neonatal death. Therefore, by the end of the session, the community develops a need-based action plan to implement in their village to try to prevent the occurrence of similar patterns of death. The action plan includes practical strategies such as arranging money and accessing transport during an emergency. The community group members make a commitment to support and implement the action plan while the community leader helps to evaluate the action plan implementation in the community [20]. Within the committee, a few take leadership and encourage the entire community to supervise and monitor the progress and overall improvement of the maternal and neonatal health situation of their village [12, 21].

### SA exploration of social and medical causes including barriers

Many studies in Bangladesh have shown that SA is effective in exploring probable medical causes of death, as well as behavioral, environmental, and social causes [22]. SA serves as an “eye opener” for the community to understand their own deficiencies while helping community health workers understand the gaps of health care services. Several studies stated that “SA reflects the delays behind a maternal death.” Furthermore, SA sensitizes the community to carefully consider their problems, misunderstandings, and delays [18]. As a result, at the end of a SA session, community people join the meeting and receive important take-way messages to prevent similar future deaths [22].

## Way forward

### Community remedial action plan and implementation (response)

Following discussion at the SA meeting, the community takes a number of decisions at the local level to address the factors responsible for a death. The World Health Organization guidelines for maternal death surveillance and response address the effective actions or responses to avert future maternal, perinatal, and neonatal deaths [4]. In the death review system in Bangladesh, SA is a key tool implemented immediately following a death along with the development of an action plan.

Many notable scenarios have been identified where we have seen that SA created a change in the behavioral pattern of a family, into a practice [12]. Nowadays, many pregnant mothers are planning the delivery of their babies in medical facilities after listening to SA meeting discussions [23]. The WHO reported a case study in Bangladesh where a mother attended a SA during her seventh month of pregnancy and following the meeting decided to have her baby delivered safely in a health facility by a skilled birth attendant [24]. Another study reported in a MDSR network, reported a young mother, carrying her first pregnancy, who came to the realization of the possible complications of childbirth as a result of attending a community SA. It was discussed that in one incident delivery by an untrained birth attendant caused postpartum bleeding in a pregnant woman in her community. Because the community did not take a decision to seek help immediately after the bleeding started, this resulted in a worsening of the condition of the mother, causing her death. From the meeting, the young mother decided to have her baby delivered by a skilled birth attendant at any cost. [12]. Likewise, SA also increased the demand of the community to seek adequate health care services from a recommended facility for mothers and newborns [12, 23, 24].

### SA to reduce maternal and neonatal mortality and reach SDG

SA of maternal and neonatal deaths is an intervention platform for discussion and interaction between the community government health workers in Bangladesh. SA is not data-driven, and no tools are used for the collection of information. This creates avenues to understand community demands, knowledge gaps, and the challenges that need to be overcome by the community [12, 13]. Through participating in SA, community members understand how to achieve change. Discussion about death in the community acts as a powerful example to know what mistakes were made and how to prevent them in the future. Responses at the grass root level could be the most effective way to prevent future maternal and neonatal deaths [25]. SA is a platform to empower the community to think, to plan, and to act, within their existing resources, towards a positive direction under an accountable framework.

In Bangladesh, SA is being implemented by the government health system. In this way, the entire intervention is focused on reaching the government mandate of achieving SDG on time [25]. National guidelines for MPDSR in Bangladesh are also being implemented. [11]. Government health care providers in all aspects and sectors are the key strength in facilitating SA; there are many proven results from health managers at all levels of the community acceptance of SA [19].

## Conclusions

By using community interaction and a participatory decision-making process, SA is a wonderful opportunity to prevent avoidable maternal and neonatal deaths.. SA is being implemented through the government’s ongoing MPDRS system in Bangladesh; therefore, at the local level, it has the opportunity to effectively address the desired SDG by 2030. Active participation of the community from the very beginning up to the end of the session builds up community accountability and encourages determination to implement their own action plans. With programs like SA, Bangladesh is on a good track to reduce maternal and neonatal morality,

## Abbreviations

LMICs: Low- and middle-income countries
MPDR: Maternal and Perinatal Death Review
MPDSR: Maternal and Perinatal Death Surveillance and Response
SA: Social autopsy
SDG: Sustainable development goal

## References

[1] Källander K, Kadobera D, Williams TN, Nielsen RT, Yevoo L, Mutebi A (2011). Social autopsy: INDEPTH network experiences of utility, process, practices, and challenges in investigating causes and contributors to mortality. Popul Health Metr.

[2] Waiswa P, Kalter HD, Jakob R, Robert E. Increased use of social autopsy is needed to improve maternal, neonatal and child health programmes in low­income countries. Bull World Health Organ. 2015:10–3. 10.2471/BLT.12.105718 PMC3370373 22690025

[3] Rao BV (2014). Social audit and verbal autopsy on maternal deaths in Prakasam district, Andhra Pradesh. J Evol Med Dent Sci.

[4] Biswas A. Maternal, newborn, child and adolescent health Maternal and Perinatal Death Review (MPDR): experiences in Bangladesh. World Health Organisation. 2015 [cited 2017 Jan 19]. p. 1–5. Available from: http://www.who.int/maternal_child_adolescent/epidemiology/maternal-death-surveillance/case-studies/bangladesh-study/en/.

[5] Biswas A, Rahman F, Halim A, Eriksson C, Dalal K (2014). Maternal and Neonatal Death Review (MNDR): a useful approach to identifying appropriate and effective maternal and neonatal health initiatives in Bangladesh. Health.

[6] Rahman KM, Olsen A, Harley D, Butler CD, Mondal D, Luby SP (2014). Kala-azar in pregnancy in Mymensingh, Bangladesh: a social autopsy. PLoS One.

[7] Baset M, Towner E, Mashreky S, Rahman A, Biswas A, Rahman F (2012). Social autopsy: a community based intervention in preventing road traffic injuries––experience from Bangladesh. Inj Prev.

[8] Arauz MJ, Ridde V, Hernández LM, Charris Y, Carabali M, Villar LÁ (2015). Developing a social autopsy tool for dengue mortality: a pilot study. PLoS One.

[9] Mashreky SR, Baset K, Rahman F, Rahman A. The social autopsy––a tool for community awareness after a drowning event. World Dorwning Conference. 2011; 1388:442. [cited 2017 Jan 19]. p. 1–5. Available from: http://www.worldconferenceondrowningprevention2011.org/SiteMedia/w3svc1092/Uploads/Documents/WCDP2011_LMIC_Baset_p65_Abstract.pdf.

[10] Baset MK, Towner E, Mashreky SR, Rahman A, Biswas A, Rahman F. Social autopsy: a community based intervention in preventing road traffic injuries––experience from Bangladesh. Injuryprev. 2012 – 040590u. Available from http://injuryprevention.bmj.com/content/injuryprev/18/Suppl_1/A205.2.full.pdf. Accessed 12 Jan 2017.

[11] Ministry of Health and Family Welfare (MoHFW). National Guideline on MPDSR 2016 [cited 2017 Jan 28]. Available from: http://www.qis.gov.bd/pdf/mpdr.pdf.

[12] Biswas A. Social autopsy as an intervention tool in the community to prevent maternal and neonatal deaths: experiences from Bangladesh social autopsy. MDSR Action Network 2016 [cited 2016 Jul 24]. p. 6–8. Available from: http://mdsr-action.net/tag/social-autopsy/.

[13] Mahmud R et al. Social autopsy triggers community response for averting maternal and neonatal death in Bangladesh. World Health Organisation. 2016. [cited 2017 Feb 24]. p. 1–5. Available from: http://www.who.int/maternal_child_adolescent/epidemiology/maternal-death-surveillance/case-studies/social-autopsy-bangladesh/en/#.

[14] Dikid T, et al. Maternal and perinatal death inquiry and response project implementation review in India. Journal of obstetrics and gynaecology of India, 2013;63(2), pp.101–107. Available at: http://www.pubmedcentral.nih.gov/articlerender.fcgi?artid=3664695&tool=pmcentrez&rendertype=abstract (Accessed 28 Jan 2015). 10.1007/s13224-012-0264-3 PMC3664695 24431614

[15] Koffi AK (2015). Social autopsy study identifies determinants of neonatal mortality in Doume, Nguelemendouka and Abong–Mbang health districts, Eastern Region of Cameroon. Journal of Global Health.

[16] Moyer CA (2017). Using social autopsy to understand maternal, newborn, and child mortality in low-resource settings: a systematic review of the literature. Glob Health Action.

[17] World Health Organization (WHO) (2016). Strengthening country capacity on maternal and perinatal death surveillance and response.

[18] The Family Initiative. Family ignorance linked to maternal deaths (Bangladesh). 2016. [cited 2017 Feb 24]. p. 1–5. Available from: https://familyincluded.com/family-ignorance-maternal-deaths-bangladesh/.

[19] Biswas A, Rahman F, Eriksson C, Halim A, Dalal K (2016). Social autopsy of maternal, neonatal deaths and stillbirths in rural Bangladesh: qualitative exploration of its effect and community acceptance. BMJ Open.

[20] Brown BJ, Oladokun RE, Osinusi K (2009). Situation analysis of the existing infant feeding pattern at the commencement of the prevention of mother to child transmission (PMTCT) of HIV programme in Ibadan. Niger J Clin Pract.

[21] Biswas A. A social autopsy of a maternal deaths explored social dilemmas in the community: findings during DFATD visits in Moulvibazar District. 2015. p. 1–2. [cited 2017 Jan 19]. p. 1–5. Available from: http://www.ciprb.org/a-social-autopsy-of-a-maternal-deaths-explored-social-dilemmas-in-the-community-findings-during-dfatd-visits-in-moulvibazar-district/.

[22] Biswas A, Halim MA, Dalal K, Rahman F (2016). Exploration of social factors associated to maternal deaths due to haemorrhage and convulsions: analysis of 28 social autopsies in rural Bangladesh. BMC Health Serv Res.

[23] Mahmud R et al. Social autopsy triggers community response for averting maternal and neonatal death in Bangladesh. World Heal Organ .2016;1–5. Available from: http://www.who.int/maternal_child_adolescent/epidemiology/maternal-death-surveillance/case-studies/social-autopsy-bangladesh/en/#. Accessed 14 Jan 2017.

[24] World Health Organization. Time to respond. Geneva; 2016. Available from: http://apps.who.int/iris/bitstream/10665/249524/1/9789241511230-eng.pdf.

[25] Biswas A (2015). Maternal and Neonatal Death Review System to improve maternal and neonatal health care services in Bangladesh.

